# Investigating the detection of parent-child relationships in early childhood: The role of partiality in resource distributions

**DOI:** 10.3389/fpsyg.2022.916266

**Published:** 2022-08-24

**Authors:** Anna Michelle McPhee, Sinamys Bagh, Mark A. Schmuckler, Jessica A. Sommerville

**Affiliations:** ^1^Toronto Early Cognition Laboratory, Department of Psychology, University of Toronto, Toronto, ON, Canada; ^2^The Laboratory for Infant Studies, Department of Psychology, University of Toronto Scarborough, Toronto, ON, Canada

**Keywords:** partiality, resource distributions, social affiliation, kinship, parent-child relationships, social inferences, social evaluations, parental investment

## Abstract

By early childhood, children possess clear expectations about how resources should be, and typically are, distributed, expecting and advocating for equal resource distributions to recipients. Moreover, recent evidence suggests that children may be able to use deviations from equality in resource distributions to make inferences about the nature of social relationships. Here, we investigated whether children use partiality in resource distributions displayed by adults toward children in third-party contexts to identify parent-child relationships, whether children anticipate preferential treatment based upon knowledge of third-party parent-child relationships, and whether children anticipate different emotional reactions to impartiality in resource distributions in parent-child interactions compared to neighbor-child interactions. Four-to seven-year-old children were presented with hypothetical vignettes about an adult character who distributed resources to two children either equally, or systematically favoring one child. By the age of 4, children used resource distribution partiality to *identify* an adult as a child’s parent, and also used these expectations to guide their anticipated emotional reactions to impartiality. By the age of 6, children were also more likely to *anticipate* partiality to be displayed in parent-child compared to neighbor-child relationships. The findings from the current study reveal that partiality in resource distributions acts as a valuable cue to aid in identifying and understanding social relationships, highlighting the integral role that resources play in children’s understanding of their social world. More broadly, our findings support the claim that children use cues that signal interpersonal investment to specify and evaluate parent-child relationships in third-party contexts.

## Introduction

Adults and children care a lot about how resources are distributed—they have expectations regarding how resources will be distributed (Ziv and Sommerville, 2017) and evaluate others based on how these individuals distribute resources (McAuliffe et al., 2017; Lucca et al., 2018). Although individuals often expect and advocate for resource distributions based on principles or norms related to fairness, such as equality, equity and merit (Schmidt et al., 2016), in the real world, sometimes resource distributions favor some individuals over others for reasons that are orthogonal to fairness (Lee et al., 2018). Specifically, previous research has illustrated that children’s expectations for equality in resource distributions are influenced by the degree of social closeness between the distributor and recipients, with expectations that the distributor will display partiality towards recipients who are familiar rather than unfamiliar to them (Olson and Spelke, 2008). More recent research suggests that the observation of partiality in resource distributions may also be used as a cue to identify the presence and nature of particular social relationships; for example, children use partiality in resource distributions to identify third-party friendships (Liberman and Shaw, 2017). Here, we investigated 4- to 7-year-old children’s abilities to use partiality in resource distributions in service of identifying parent-child relationships in third-party contexts. We also examined whether children were able to make the reverse inference: to use knowledge of parent-child relationships to guide their expectations for partiality, and whether these expectations were used to guide their anticipated emotional reactions to impartiality.

## Children’s expectations and evaluations of resource distributions

Research suggests that within the first few years of life (Geraci and Surian, 2011; Schmidt and Sommerville, 2011; Sloane et al., 2012; Ziv and Sommerville, 2017), infants begin to develop egalitarian expectations for how resources should be distributed: they expect resources to be distributed equally between recipients. For example, using a violation-of-expectation paradigm, researchers found that infants as young as 15 months of age looked longer at displays in which a distributor allocated resources unequally, compared to equally, amongst recipients (Schmidt and Sommerville, 2011). More recent work has extended the developmental onset of this ability to 10–12 months of age (Meristo and Surian, 2014; Ziv and Sommerville, 2017). Importantly, infants do not appear to hold expectations for equality in situations lacking social context (i.e., when only the outcomes of resource distributions were shown, devoid of any recipients; Schmidt and Sommerville, 2011), nor for situations involving inanimate objects as recipients instead of human actors (Sloane et al., 2012; Ziv and Sommerville, 2017). These expectations were also absent in contexts in which partiality in resources were ‘found/revealed’ by a distributor, rather than deliberately created as a sign of partiality displayed by the distributor towards one recipient over another (Sloane et al., 2012). Thus, there is evidence to suggest that before the second birthday, children develop equality norms for resource distributions and selectively apply them to social contexts.

Moreover, studies have illustrated that children’s expectations for equality become increasingly robust across the preschool period, such that when given the opportunity to distribute resources themselves in a third-party task, 3-year-old children tend to display an egalitarian preference and distribute resources equally between two recipients (e.g., Olson and Spelke, 2008; Baumard et al., 2012; Shaw and Olson, 2012). Children’s preferences for egalitarianism become quite sophisticated across early to mid-childhood, such that by the age of 6, children would rather discard an extra resource than to distribute it and create an unequal distribution of resources (Shaw and Olson, 2012). Together, these studies illustrate that children have a robust understanding of distributive fairness and in the absence of background information, expect resources to be distributed equally amongst recipients.

Of course, equality is not the only norm that guides resource distributions: children are also aware of other fairness norms that influence resource distributions, such as need (Paulus, 2014) and merit (Baumard et al., 2012), and adjust their expectations for equality in resource distributions accordingly. For example, Paulus (2014) found that children as young as 5 altered their expectations for how stickers should be distributed in a third-party resource distribution task as a function of whether the recipient was described as ‘rich’ (i.e., had an abundance of stickers) or ‘poor’ (i.e., had minimal stickers), with more resources being distributed towards the latter compared to the former. Moreover, a study by Baumard et al. (2012) illustrated that preschool-aged children distributed more resources towards a hard-working actor (i.e., by giving them a bigger rather than smaller cookie and/or by allocating more resources [cookies] to them) compared to a lazy agent who did not contribute as much to task completion. In sum, although children hold egalitarian preferences, preschoolers are able to take various principles related to fairness into account when determining how resources should be distributed.

Critically, sometimes resource distributions are guided by reasons orthogonal to fairness, such as the type of relationship present between the distributor and recipient. For example, Lee et al. (2018) found that although preschool-aged children demonstrated egalitarian preferences in a third-party resource distribution task between in-group and out-group members, in situations in which there was an unequal number of resources to be distributed, participants preferentially allocated the extra resource to an in-group member. More specifically, aside from group membership, evidence suggests that children expect the nature of the interpersonal social relationship between the distributor and recipients to influence resource distributions. As illustrated in a study by Olson and Spelke (2008), 3.5-year-old children modified their baseline fairness expectations to anticipate advantageous resource distributions to be given to familiar (i.e., siblings and friends) rather than unfamiliar (i.e., strangers) individuals in a third-party resource distribution task. Paulus and Moore (2014) found similar evidence that children’s expectations for sharing of resources differed as a function of the recipient’s identity in both first-person and third-party contexts: specifically, 4- and 5-year-old participants were more likely to select an equitable distribution of resources (1:1 ratio) in hypothetical contexts that involved a protagonist character (or themselves) sharing with a friend, and more likely to select an unequitable distribution of resources (2:0 ratio in favor of the protagonist) when sharing with a disliked peer. Moreover, evidence suggests that children cannot only anticipate the distribution of resource distributions based on social relationships but can also make the reverse inference: 4-year-old children inferred that a distributor and target child were friends after seeing the distributor give 3 resources (e.g., colorful erasers) to the target child compared to 2 to another child (Liberman and Shaw, 2017). Yet, despite these findings, whether children specifically use partiality in resource distributions as a cue to kinship relationships, and particularly parent-child relationships, remains a largely unexplored topic.

## Identifying kinship relationships

Parent-child relationships are particularly important in development as they are essential for children’s well-being and survival (Trivers, 1974; Kaplan et al., 2000). Parents are responsible for providing their children with access to food and water to help ensure the well-being of their child (Keller, 2000). Evidence shows that parents selectively invest in their children over and above other individuals (e.g., Anderson et al., 1999); for example, adults reported more willingness to serve jail time or donate a kidney if the benefits of doing so were directed towards their own child compared to a niece/nephew or a friend’s child (Antfolk et al., 2017). Critically, these unique features of parent-child relationships may provide cues to outside observers. Given the prevalence and fundamental role that parental investment plays in children’s own lives, it is possible that the observation of parental investment in third-party contexts may be used as a cue to identify parent-child relationships. Consistent with this claim, research with adult populations has shown that individuals not only rely on obvious cues, such as facial similarity (Kaminski et al., 2009), to aid in kinship detection, but also use social cues, such as the observation of maternal investment, to do so (Lieberman et al., 2007). For example, undergraduate students reported that the observation of maternal perinatal association (e.g., a mother breastfeeding an infant) was the strongest cue to aid in first-person sibling kinship detection while growing up (Lieberman et al., 2007).

## Overview of current study

Given that one of the most fundamental types of parental investments is the allocation of resources, such as food, to children (Keller, 2000), and given findings showing that parents regularly favor their children over other children in various real-world forms of resource allocations (Anderson et al., 1999; Berghel, 2020; Davies, 2021) we theorized that the observation of an adult preferentially allocating food to one child over another may serve as a valuable cue for children to identify parent-child relationships in third-party contexts.

In our study, participants between the ages of 4- to 7-years-old were presented with hypothetical vignettes in which an animated adult character interacted with two children in a resource distribution paradigm. In the Kinship task, participants viewed the adult distribute resources in either an advantageous (3:1 ratio favoring the protagonist) or equitable (2:2 ratio neither favoring nor hindering the protagonist) manner and were asked to determine whether the adult was or was not the mother of the protagonist; we predicted that children would use the observation of partiality as a cue to identify third-party parent-child relationships. In the Resource Distribution task, the adult character was identified as either the mother or neighbor of the protagonist character and participants were asked to determine how the adult would allocate the resources to the two children (advantageous [3:1 ratio favoring the protagonist], equitable [2:2 ratio neither favoring nor hindering the protagonist] or disadvantageous [1:3 ratio not favoring the protagonist] resource distribution); we anticipated that participants would be more likely to anticipate an equitable distribution of resources when the adult was identified as the neighbor compared to mother. In the Emotion task, participants were asked to determine how the protagonist character would feel if the adult (identified as either the mother or neighbor) allocated the resources in an equitable manner; we hypothesized that an equitable distribution of resources would lead participants to anticipate less positive emotions by the protagonist child if impartiality towards them was displayed by a parent, compared to a neighbor. Together, these patterns of findings would provide support for the hypothesis that partiality in resource distributions is not only important for identifying social relationships such as friendships (i.e., Liberman and Shaw, 2017), but also serve as a cue to identifying parent-child relationships in third-party contexts. Moreover, the present study investigates whether children use the link between partiality and parent-child relationships bidirectionally (i.e., partiality to infer parent-child relationships, and parent-child relationships to infer partiality), and also whether children can anticipate emotional reactions to impartiality displayed by either a parent or neighbor.

Four- to five-year-olds performance was compared to 6- to 7-year-olds’ to examine the developmental trajectory of these abilities across early childhood. These particular age groups were selected given previous research illustrating that children as young as 4 use the observation of partiality to infer friendship between two peers (Liberman and Shaw, 2017), and by the age of 5, use knowledge of familial relationships (e.g., sibling relationships) to anticipate selective displays of prosocial behavior (Spokes and Spelke, 2016).

## Methodology

### Participants

Children between the ages of 4- to 5- (*N* = 34, *M* = 60 months 9 days; *SD* = 6 months and 24 days; range: 50 months 14 days to 71 months 17 days; 13 participants were female), and 6- to 7-years-old (*N* = 34, *M* = 84 months 1 day; *SD* = 7 months and 5 days; range: 72 months 10 days to 94 months 16 days; 16 participants were female) were recruited from a database maintained by a public university situated in a large city in North America. An additional 9 children were tested but were not included in the final sample due to parental interference (*n* = 2), technological errors (*n* = 2), experimenter error (*n* = 3) and failure to complete the experiment (*n* = 2). The average self-reported annual household income was approximately $145,139 CAD; however, only 36 out of 68 parents (53%) disclosed this information. Although no formal demographic information about ethnicity was obtained, the sample was generally diverse and representative of the city in which the testing originated.

Prior to the commencement of the study, parents read and signed an online version of the consent form *via* Qualtrics (a secure online survey software program). Child verbal assent was also sought. The experimental protocol was in accordance with the 1964 Declaration of Helsinki and was approved by the university ethics board.

### Procedure

Due to the COVID-19 outbreak, data collection took place online *via* Zoom while isolation procedures for the pandemic were in effect. Prior to the commencement of experimental sessions, parents were asked to set their computer up in a distraction-free environment with a secure internet connection and a fully charged computer battery. During the experiment, the experimenter’s screen was shared with the participants so that they were able to view both the stimuli and the experimenter throughout the session. To help ensure consistency across participants, specific instructions regarding how to set up the webcam (e.g., having the webcam angled at 60 degrees so that the participants’ responses were visible and audible) and the Zoom call (e.g., hide ‘Self-View’, turn off ‘Side-by-Side Mode’ and place the video of the experimenter in the bottom right corner of the screen) were reviewed with the parents at the beginning of the session. The children’s responses were coded live, and the videotaped sessions were independently coded by a naïve coder. Parents were asked to not guide their child’s answers in any way or repeat the instructions. If the participants did not understand or hear the question, the experimenter would repeat the information as necessary.

The experiment consisted of three separate tasks that were delivered in a consistent order: (1) the Kinship task, (2) the Resource Distribution task and, (3) the Emotion task. Given that this study was one of the first studies our laboratory conducted online through Zoom, we were unsure as to whether or not children would be sufficiently attentive to complete all tasks. Therefore, we chose a fixed order for the tasks to ensure that we would have an adequate sample size for at least a subset of the tasks (i.e., those at the start of the procedure).

Each task featured animated scenarios that were created using online animation software (Pixton (2021) ^1^ for the Kinship task, and Notability, Ginger Labs Inc (2021) ^2^ for the Resource Distribution task and Emotion task). The Kinship and Resource Distribution tasks each consisted of two different trials (within-subjects design) and the Emotion task consisted of one trial (between-subjects design). Although the tasks were presented in a fixed order, the order in which the trials were administered within each task was counterbalanced. The gender of the protagonist character in all of the scenarios was matched to the participant.

**Figure 1:**
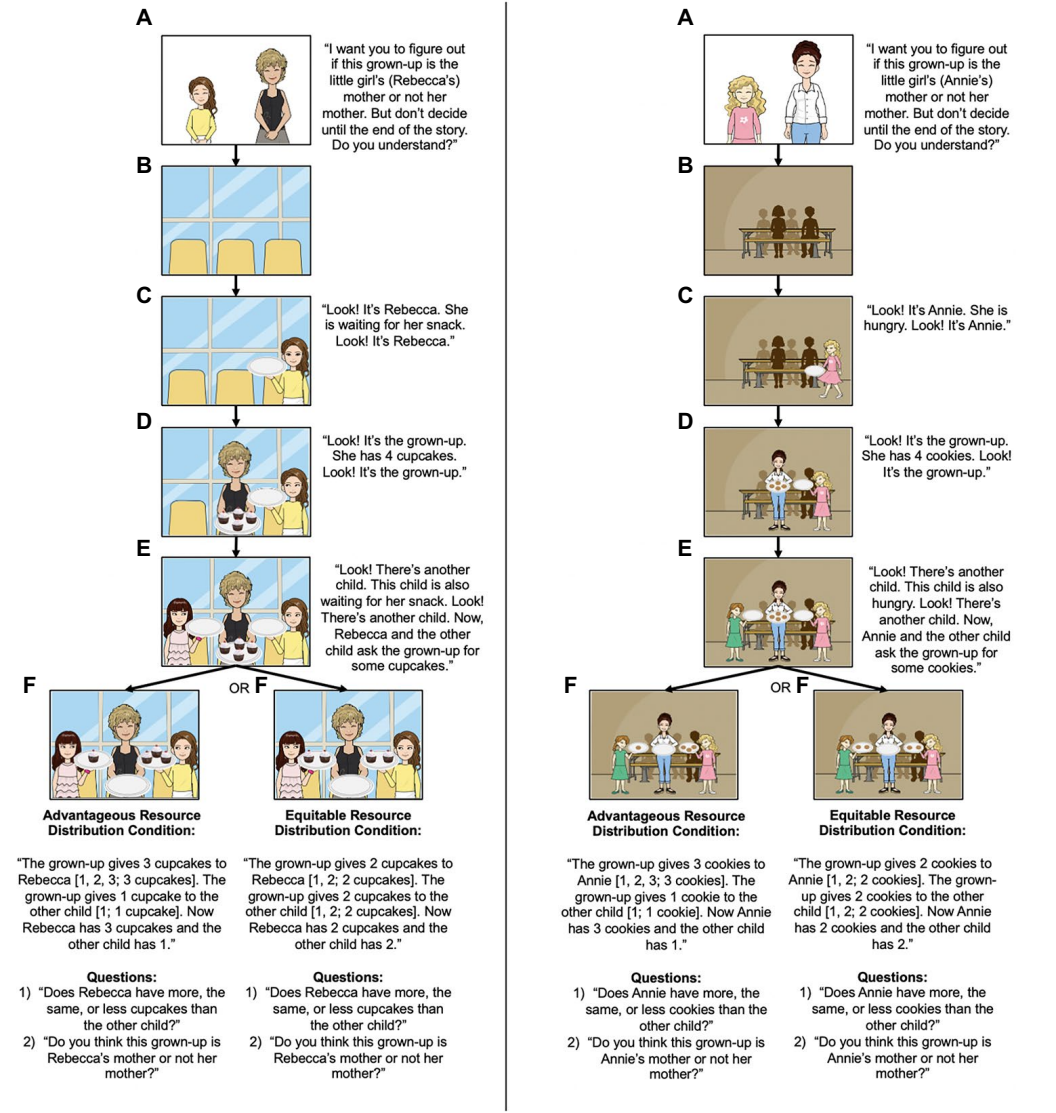
The Kinship Task. Participants were read two comic book scenarios in which they observed an adult character allocate resources. In one scenario, the adult distributed the resources fairly (the equitable resource distribution condition**—**2:2 ratio), and in the other, the adult distributed the resources unfairly (the advantageous resource distribution condition**—**3:1 ratio in favor of the protagonist character). After each scenario, participants were asked to determine whether or not the grown-up was or was not the mother of the protagonist character. The stories were told following an **(A)**–**(F)** sequence.

The Kinship task, shown schematically in Figure 1, investigated whether 4- to 5-year-olds and 6- to 7-year-olds use information about how resources are distributed to infer kinship (or lack thereof) in adult-child interactions. During this task the experimenter narrated comic book scenarios that depicted an adult character distributing resources (cookies or cupcakes) to two child characters. In one scenario, the adult character distributed the resources in an equitable manner between the two children (the equitable resource distribution condition, 2:2 ratio), whereas in the other scenario, a different adult character distributed the resources unequally in favor of the protagonist (the advantageous resource distribution condition, 3:1 ratio).

Following each scenario, participants were first asked comprehension questions about the quantity of resources that each child character received (e.g., “Did Rebecca get more, the same or less cupcakes than the other child?”) to determine whether or not the participants understood and encoded the key manipulation of the scenario. If children responded incorrectly, the experimenter would remind the children of how the cupcakes were distributed (e.g., for the advantageous resource distribution condition the experimenter said, “Remember, the other child has 1 cupcake and Rebecca has 3 cupcakes. So does Rebecca have less, the same, or more cupcakes than the other child?”). If children still responded incorrectly, the experimenter would tell the participants the correct answer (e.g., for the advantageous resource distribution condition the experimenter said, “Actually, I think Rebecca has more cupcakes than the other child because the other child has 1 cupcake and Rebecca has 3 cupcakes. So, I think Rebecca has more.”).^3^

Next, participants were asked the key test question about the type of relationship they believed existed between the adult and child protagonist: specifically, the participants were asked to determine if the adult was, or was not, the mother of the protagonist.^4^ We anticipated that children would be more likely to identify the adult as the child’s mother when the adult distributed the resources in an advantageous, compared to equitable, manner to the protagonist child.

**Figure 2:**
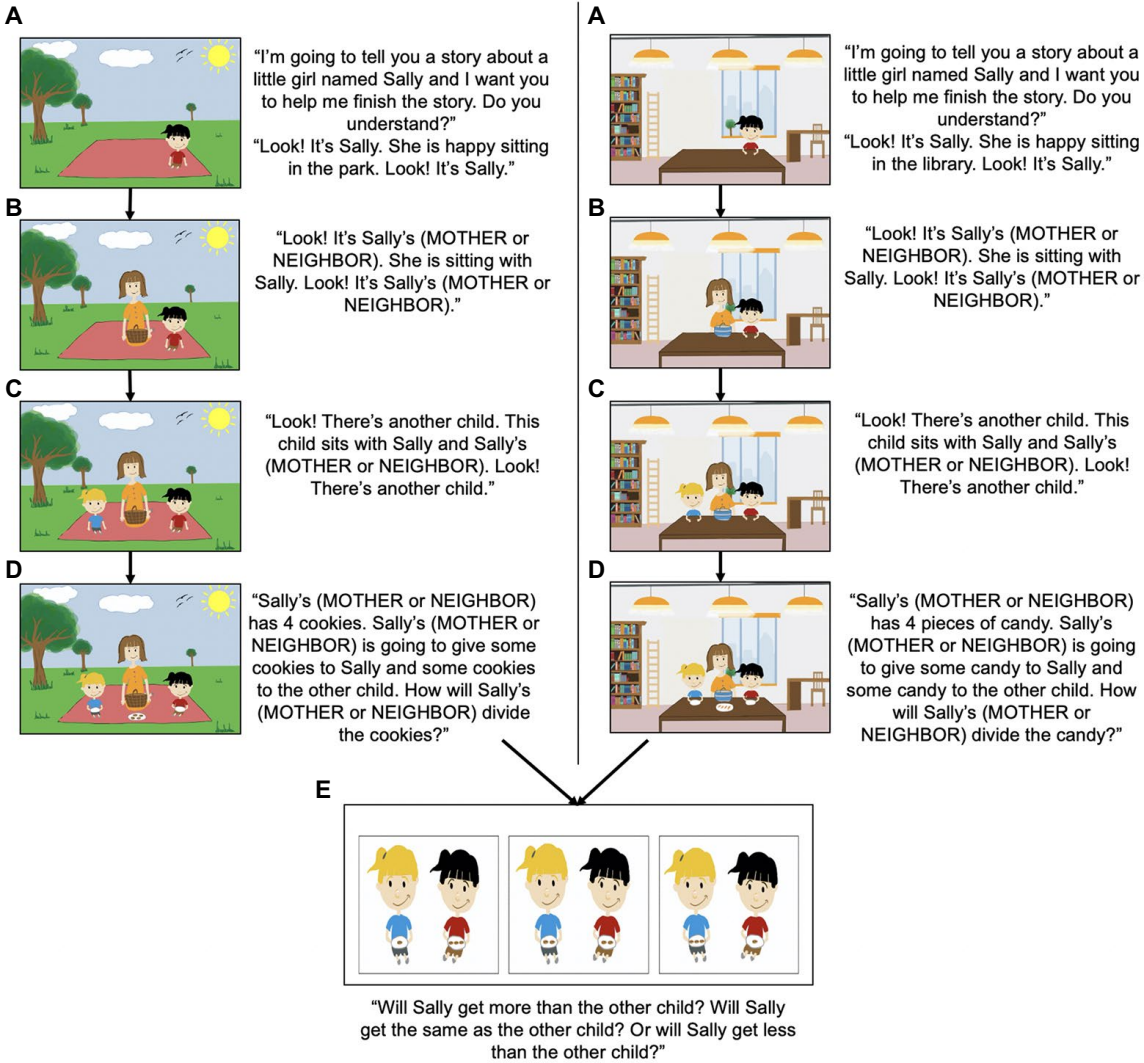
The Resource Distribution Task. Participants were read two storybook scenarios about a protagonist child, an adult (either the protagonist’s mother or neighbor) and another child. Participants were asked to decide how the adult would distribute the resources (advantageous, equitable, or disadvantageous to the protagonist). The stories were told following an **(A)**‑**(E)** sequence.

The Resource Distribution task, shown schematically in Figure 2, investigated whether children use kinship cues to help guide their expectations about how resources will be distributed. Participants were read two different storybook scenarios about an adult character and two child characters; the key variation between the scenarios was the identity of the adult, specifically whether the adult was identified as the protagonist’s mother or neighbor. Each story began by introducing the protagonist, followed by the adult—who was labeled as either the protagonist’s mother or neighbor—and then another child. After all of the characters were introduced, the adult ‘discovered’ a set of resources (cookies or candy) with the intention of distributing the resources between the two children. Participants were asked to determine how the adult would allocate the resources by selecting one of three options, such that the protagonist could receive: (1) fewer resources than the other child (a disadvantageous resource distribution; 1:3 ratio not favoring the protagonist), (2) an equal amount of resources as the other child (an equitable resource distribution; 2:2 ratio neither favoring nor hindering the protagonist) or (3) more resources than the other child (an advantageous resource distribution; 3:1 ratio, favoring the protagonist). The response option locations on the screen were counterbalanced across participants. We predicted that participants would be more likely to indicate that the adult would allocate the resources equally when she was the child’s neighbor than when she was the child’s mother.

**Figure 3:**
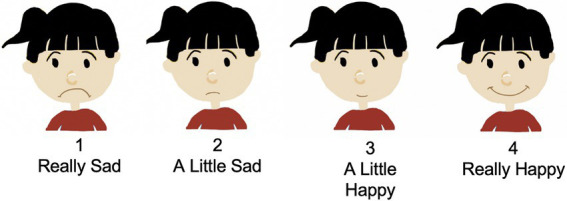
The Emotion Task. Modified 4-point Likert scale of emotion used in the Emotion task. Responses ranged from Really Sad (1) to Really Happy (4).

The Emotion task, shown schematically in Figure 3, was an extension of the Resource Distribution task; it consisted of asking participants to determine how the protagonist character would feel (using a modified 4-point Likert scale of emotion; original 7-point Likert scale developed by Andrews and Withey, 1976) if the adult distributed the resources in an equitable manner to the protagonist and the other child.^5^ Children were only asked this question for the final scenario with which they were presented with in the Resource Distribution task, such that half of the children were asked this question when the adult was the mother and half were asked this question when the adult was the neighbor (between-subjects manipulation). If participants had selected an equal resource distribution in the previous task, they were immediately asked how the protagonist character would feel. If participants had selected a different option (a disadvantageous or advantageous resource distribution), they were asked to imagine how the protagonist would feel if the distribution had been equal.

Recall that in the Resource Distribution task, we predicted that participants would expect equality in resource distributions more frequently in the neighbor compared to the mother condition. In the Emotion task, we further predicted that in a hypothetical scenario in which those expectations were not met (such that the mother distributed the resources equally), that the participants would anticipate less positive emotional reactions in the mother compared to the neighbor condition.

### Coding

The participants’ responses were coded live by the experimenter and the videotaped recordings of the sessions were coded independently by a reliability coder. Any discrepancies were settled by a third coder. Three participants’ experimental sessions were not recorded at the request of the parent, and as such, reliability checks could not be performed on these data. The first answer that participants provided was coded.

The inter-rater agreement was 99.3% for the Kinship task, 99.3% for the Resource Distribution task, and 100% for the Emotion task.

## Results

### Kinship task and resource distribution task

Given the within-subjects nature of the experimental design, the desire to compare across age groups, as well as the binary and ordinal nature of the data for the Kinship and Resource Distribution tasks respectively, we began with omnibus generalized estimating equation (GEE) modelling to analyze participants’ forced-choice responses, followed by the pre-registered statistical analyses.^6^

In the Kinship task, we were interested in examining whether children use partiality in resource distributions to infer parent-child kinship relationships and if so, whether this ability varied as a function of age group. The data were subject to a GEE to examine the impact of condition (advantageous versus equitable resource distribution), age group (4- to 5-year-olds versus 6- to 7-year-olds) and task order (advantageous versus equitable resource distribution presented first) on participants’ responses. Condition was a significant predictor of how participants responded, 𝓍^2^ (1, *N* = 68) = 7.05, *p* = 0.008; across all age groups, 72% of participants identified the adult as the mother of the protagonist after viewing an advantageous resource distribution compared to only 50% after viewing an equitable resource distribution. The analysis revealed a main effect of age group, 𝓍^2^ (1, *N* = 68) = 5.07, *p* = 0.02, such that 71% of 4-to 5-year-olds and 51% of 6-to 7-year-olds identified the adult as the mother of the protagonist (regardless of condition). No main effect of task order, 𝓍^2^ (1, *N* = 68) = 0.02, *p* = 0.88, emerged. Moreover, no significant interaction of condition by age group emerged, 𝓍^2^ (1, *N* = 68) = 0.08, *p* = 0.78. These findings suggest that 4- to 7-year-old children were more likely to identify the adult as the mother after viewing an advantageous compared to equitable resource distribution. Moreover, these results show that 4- to 5-year-olds display relatively high assumptions of parenthood regardless of adult behavior, in comparison to older children, perhaps due to the greater presence of parents in the lives of younger than older children.

**Figure 4:**
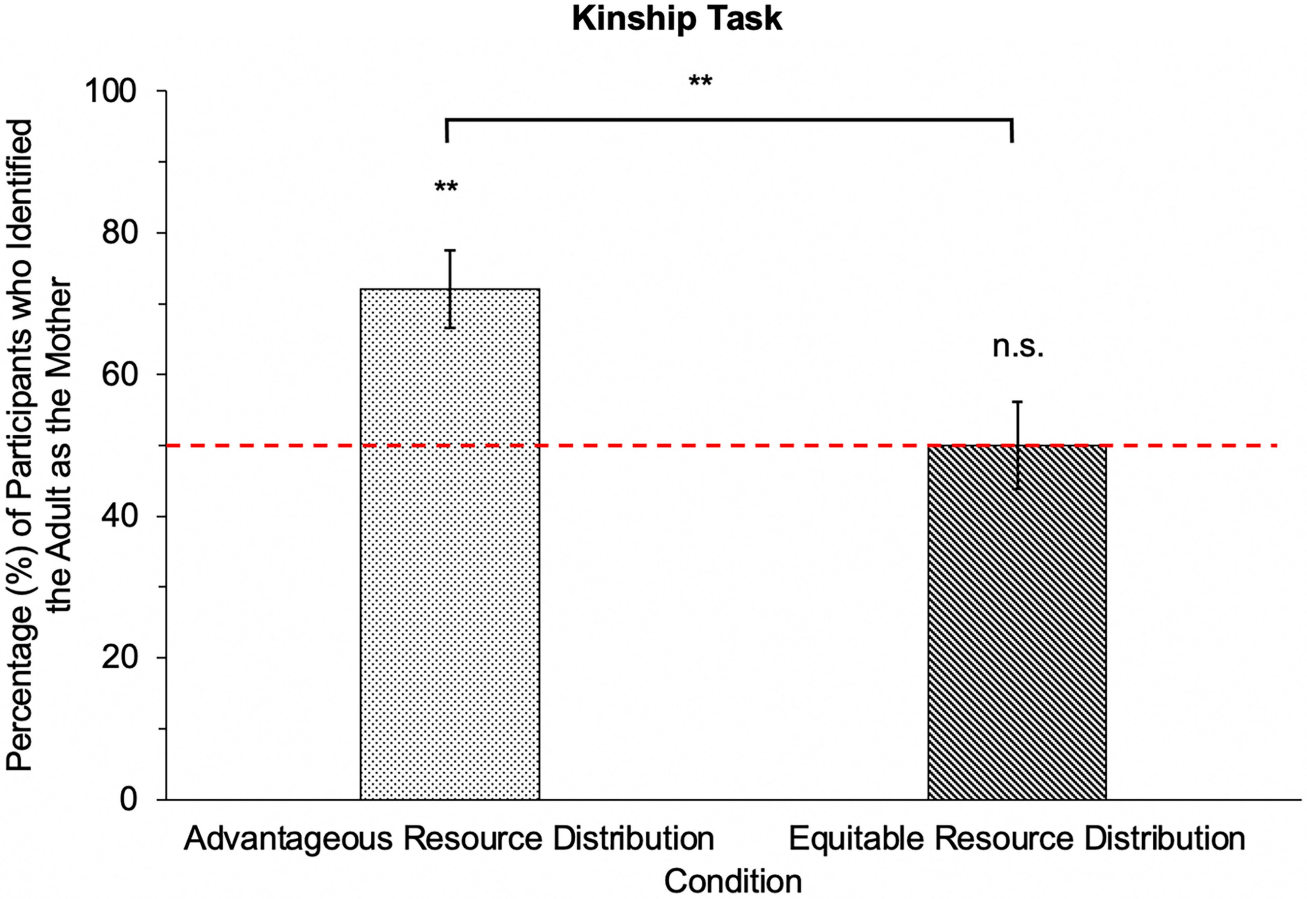
Responses on the Kinship Task. Figure 4 represents participants’ responses on the Kinship task and the percentage of participants who identified the adult character as the mother of the protagonist after viewing advantageous and equitable resource distributions. Binomial tests were conducted to examine whether participants’ identification of the adult as the mother differed from chance (dotted red line) in the advantageous and equitable resource distributions. Error bars represent standard error of the mean. **p < 0.01.

Following our pre-registered analytic plan, we compared participants’ responses further by investigating whether children used advantageous resource distributions to infer that the adult was the child’s mother at rates above chance; this data is presented in Figure 4. Binomial tests revealed that participants identified the adult as the mother of the protagonist significantly above chance in the advantageous resource distribution condition (*p* < 0.001); their responses did not significantly differ from chance in the equitable resource distribution condition (*p* = 1.00). Together, these findings suggest that children as young as 4-years-old use partiality in resource distributions to infer parent-child kinship relationships in third-party contexts.

Originally for the Resource Distribution task, we anticipated and pre-registered using a non-parametric logistic regression to analyze the results of the Resource Distribution task, however, for a number of reasons (e.g., the desire to mirror the analyses between tasks, the ordinal nature of the data, and the repeated measures design), a GEE analysis was used to examine whether condition (adult identity: protagonist’s mother or neighbor), age group (4- to 5-year-olds versus 6- to 7-year-olds) and task order (the mother or neighbor conditions presented first) influenced how participants expected the adult to distribute the resources to the two child characters.^7^

**Figure 5:**
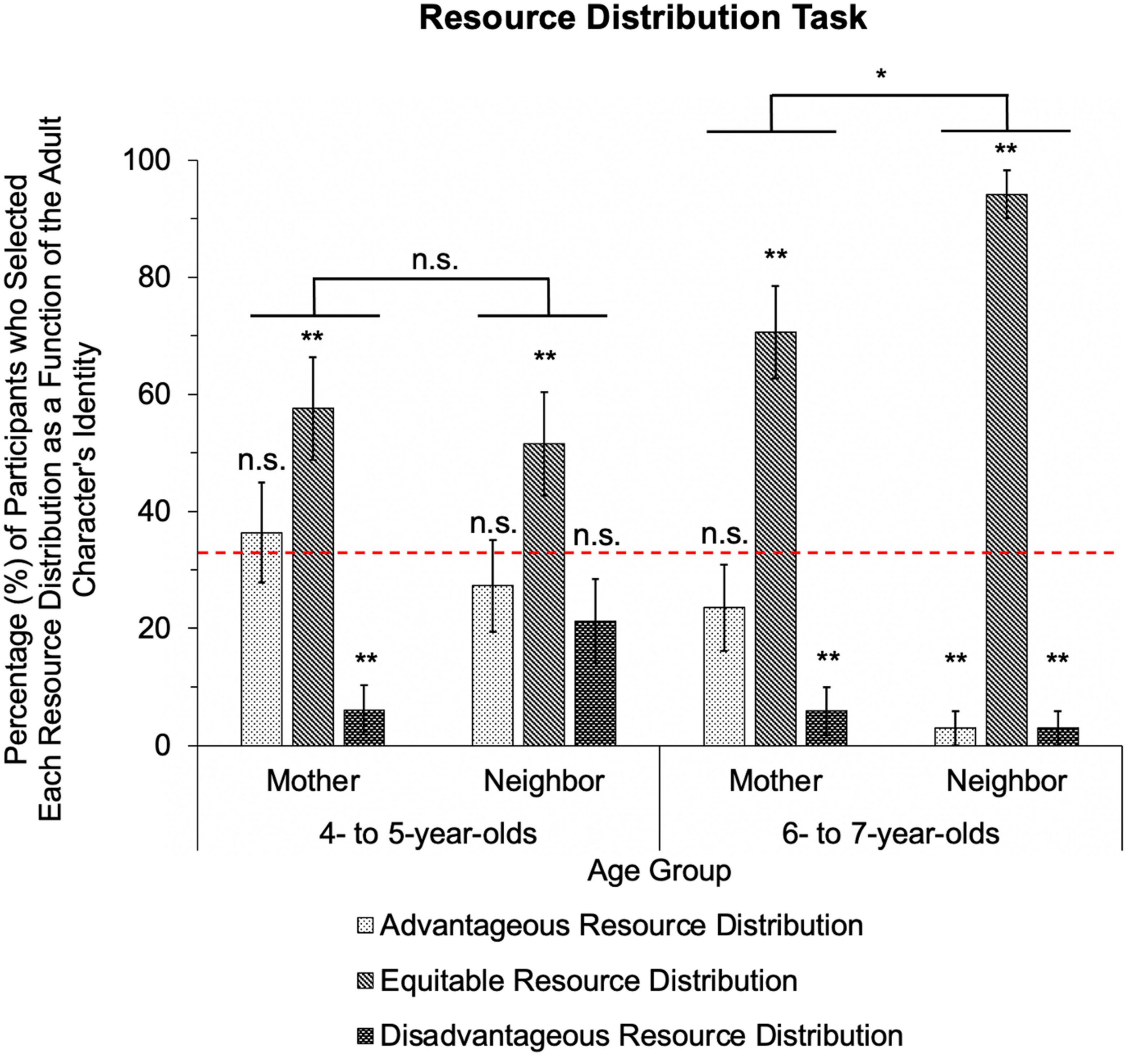
Responses on the Resource Distribution Task. Figure 5 represents participants’ responses on the Resource Distribution task and the percentage of participants who anticipated each distribution type as a function of adult identity and as a function of age groups. Error bars represent standard error of the mean. *p < 0.05; **p < 0.01.

A marginal effect of condition emerged, 𝓍^2^ (1, *N* = 67) = 2.81, *p* = 0.09. A main effect of age group emerged, 𝓍^2^ (1, *N* = 67) = 9.96, *p* = 0.002. Six-to seven-year-olds selected the equitable distribution 82% of the time, the advantageous distribution 13% of the time, and the disadvantageous distribution 4% of the time. The 4-to 5-year-olds selected the equitable distribution 55% of the time, the advantageous distribution 32% of the time, and the disadvantageous distribution 14% of the time. These findings are consistent with prior work that illustrated that children generally have increasing expectations for equality in resource distributions across these age groups (Olson and Spelke, 2008; Shaw and Olson, 2012). The main effects of condition and age were further qualified by a significant condition by age group interaction, 𝓍^2^ (1, *N* = 67) = 7.19, *p* = 0.007; this interaction appears in Figure 5. No main effect of task order, 𝓍^2^ (1, *N* = 67) = 0.42, *p* = 0.52, emerged. To investigate the effect of condition in each of our two age groups, separate follow-up GEE analyses were conducted for the 4-to 5-year-old and 6-to 7-year-old age groups. In the 4-to 5-year-old age group, condition was not found to be a significant predictor of children’s responses, 𝓍^2^ (1, *N* = 33) = 1.95, *p* = 0.16. In contrast, condition was found to be a significant predictor of participants’ responses in the 6-to 7-year-old age group, 𝓍^2^ (1, *N* = 34) = 5.52, *p* = 0.02.

Participants’ responses on the Resource Distribution task were compared to chance (0.33) for each condition and age group using binomial tests. For the mother condition, the 4- to 5-year-old participants’ selections did not significantly differ from chance when selecting the advantageous (*p* = 0.73) resource distribution option, but were significantly above chance (*p* = 0.001) and below chance (*p* < 0.001) for the equitable and disadvantageous resource distribution options, respectively. For the neighbor condition, the 4- to 5-year-olds’ selection did not significantly differ from chance when selecting the advantageous resource distribution (*p* = 0.31), nor the disadvantageous resource distribution (*p* = 0.10), but did significantly differ from chance when selecting the equitable resource distribution (*p* = 0.008). For the mother condition, the 6- to 7-year-olds’ selection of the advantageous resource distribution (*p* = 0.16) did not significantly differ from chance, but was significantly above chance when selecting the equitable resource distribution (*p* < 0.001) and significantly below chance when selecting the disadvantageous resource distribution (*p* < 0.001). For the neighbor condition, the 6- to 7-year-olds’ selection of the advantageous resource distribution (*p* < 0.001) and disadvantageous resource distribution (*p* < 0.001) were significantly below chance, while their selection of the equitable resource distribution (*p* < 0.001) was significantly above chance.

### Emotion task

Recall that children’s performances on the Emotion task were scored on a continuous 4-point Likert scale, and as such, we used a between-subjects univariate analysis of variance (ANOVA) to examine whether participants expected less positive emotions when the adult was identified as the mother compared to the neighbor.^8^

**Figure 6:**
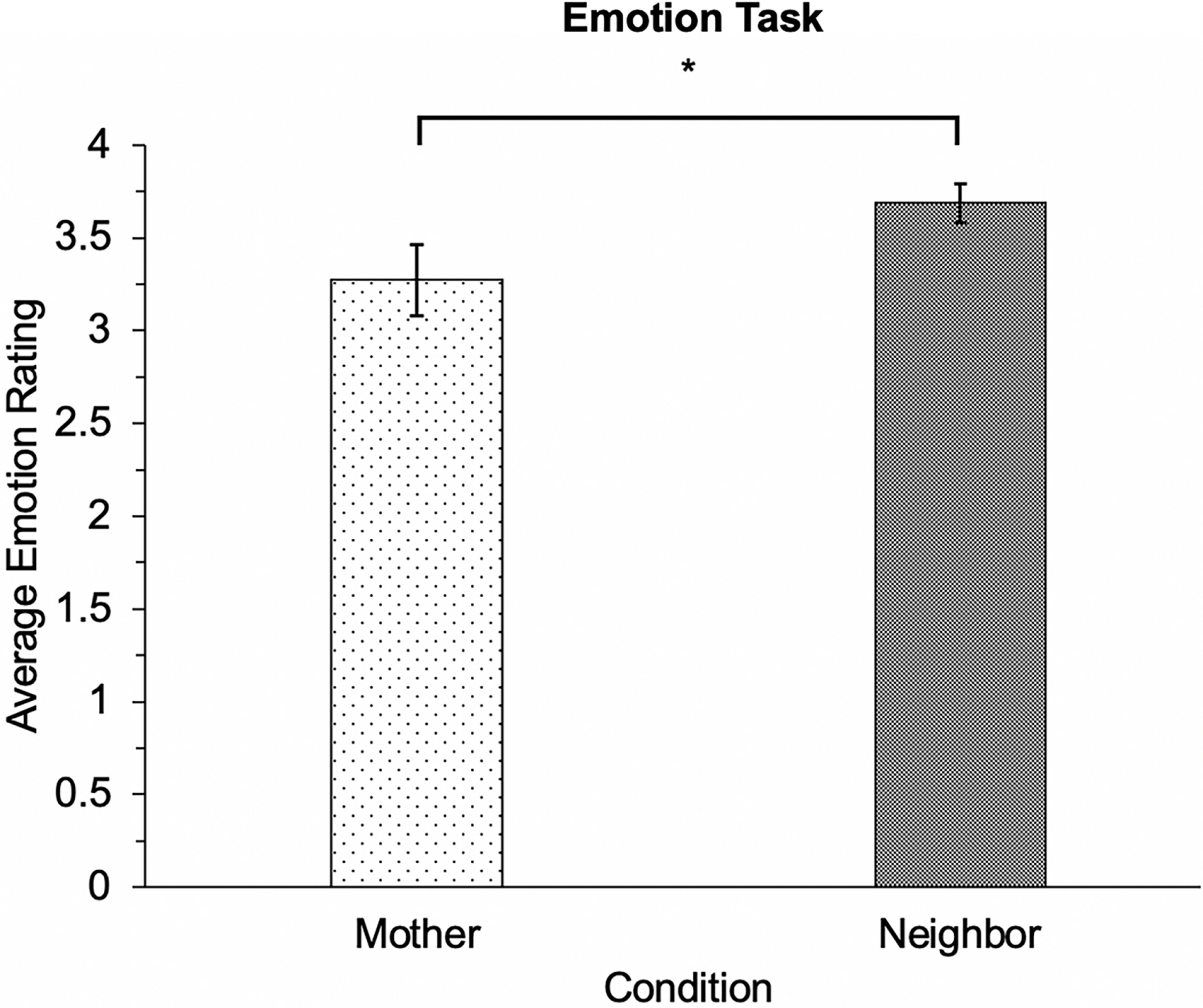
Responses on the Emotion Task. Figure 6 represents the average ratings on a modified 4-point Likert scale of emotion of how the participants anticipated the protagonist would feel if the adult, either the mother or neighbor, distributed the resources fairly between the two child characters. Error bars represent standard error of the mean. *p < 0.05.

A 2 (condition-adult identity: protagonist’s mother or neighbor) × 2 (age group: 4- to 5-year-olds versus 6-to 7-year-olds) factorial ANOVA was conducted to examine whether condition influenced how the participants anticipated the target child would feel (on a 4-point Likert scale) if they received an equitable resource distribution. A main effect of condition emerged, *F* (1, 64) = 4.24, *p* = 0.04, *n_p_*^2^ = 0.06, such that participants anticipated that the equitable distribution of resources would result in less positive emotions if the adult was the protagonist’s mother (*M* = 3.27; *SE* = 0.19) as opposed to their neighbor (*M* = 3.69; *SE* = 0.10); this effect is shown in Figure 6. There was no main effect of age group, *F* (1, 64) = 2.68, *p* = 0.11, *n_p_*^2^ = 0.04, nor a condition by age group interaction, *F* (1, 64) = 1.53, *p* = 0.22, *n_p_*^2^ = 0.02.

Thus, across all ages, participants anticipated that the target character would have less positive emotions if the mother, as opposed to neighbor, distributed the resources in an equitable manner.

## Discussion

### The developmental trajectory of children’s ability to identify parent-child relationships in third-party contexts

The findings from the current study suggest that 4 -to 7-year-old children link particular kinds of social relationships, and in this case parent-child relationships, to partiality. Children as young as 4 use partial resource distributions to identify an adult as a child’s parent versus not their parent. Similarly, children as young as 4 anticipated that a target child would feel less positively about an equitable resource distribution that was performed by the child’s parent versus the child’s neighbor. Together, these findings support the claim that children can use partiality in resource distributions by adults to identify third-party parent-child relationships. Moreover, these findings suggest that children expect parents to engage in partial resource distributions toward their children, to a greater extent than non-parents, and that children recognize the emotional consequences of impartial resource distributions by parents toward their children.

The findings from the current study indicated that children’s expectations for how resources would be distributed differed for the mother and neighbor conditions for participants aged 6 and older. However, it is important to point out that although 4- and 5-year-olds’ expectations for resource distributions made by a mother versus neighbor did not vary as a whole, children in the mother condition anticipated a disadvantageous distribution toward the target child at levels significantly below chance, whereas those in the neighbor condition anticipated a disadvantageous distribution toward the target child that did not differ from chance, suggesting an emerging ability to appreciate that the manner in which a mother or neighbor may distribute resources to a child may vary based on the social relationship. Perhaps more critically, in this task children showed a prevailing tendency to anticipate that the adult character would distribute resources equally between the two recipient children. It is likely that this pattern of results reflects children’s increasing tendency to expect resource distributions, broadly construed, to follow equality norms (Olson and Spelke, 2008; Shaw and Olson, 2012). In addition, other task differences between the Kinship task and Resource Distribution task may have driven children’s differential performance across the tasks; namely, in the Kinship task, children were provided with two response options (i.e., mother versus not mother), whereas in the Resource Distribution task, children had to decide between three options (i.e., equal distribution, advantageous distribution favoring the protagonist child, or disadvantageous distribution hindering the protagonist child). Future work should attempt to more closely match these tasks to isolate the reasons for the decalage in performance across the tasks.

Moreover, it should be noted that the current paradigm may have *underestimated* children’s ability to use partiality to infer kinship. Recall that the protagonist character was always introduced first in each scenario, and as such, participants may have used this information to envision other reasons for why the protagonist child was the recipient of an advantageous distribution of resources. For example, children might assume that the first child received more food because they were hungrier than the second child. This possibility would align with previous research illustrating that first possession heuristics are used by preschoolers to guide their understanding of social situations and ownership (Friedman and Neary, 2008). Thus, future work should counterbalance the order in which the protagonist child (i.e., the child who receives the advantageous distribution) appears in the story.

Furthermore, while we investigated children’s inferences about the relationship between the recipient of more (versus fewer) resources and the adult, another interesting question for future work concerns the inferences that children may have made about the relationship between the non-favored child and the adult. Future work can directly seek to characterize these inferences.

### The importance of identifying parent-child relationships

Irrespective, as a whole these initial findings open the door to investigating the developmental origins of the ability to identify parent-child relationships at earlier ages. Given the central role that parents play in the lives of children, and given that parental partiality towards their own children is a defining feature of parent-child relationships (e.g., Trivers, 1972), it may be the case that the origins of this ability can be traced back to earlier in life. Evidence suggests that even infants possess expectations around caregiving and caregivers that permeate their event representations; for example, infants possess expectations that caregivers will be responsive to infant distress signals in third-party contexts (Johnson et al., 2007; Jin et al., 2018). These findings raise the possibility that the ability to identify particular types of caregiving relationships—specifically parent-child relationships—may be present in infancy. Moreover, work indicating that infants are finely attuned to resource distribution events (Ziv and Sommerville, 2017), and understand at least some of the circumstances under which distributors may vary from equality in resource distributions (Enright et al., 2017), suggest that infants may draw on partiality in resource sharing to specify parent-child relationships. Future work can address this question.

### Children’s understanding of the factors influencing resource distributions

The findings from the current study help to inform theories regarding children’s expectations for partiality in resource distributions more broadly. These findings are in accordance with previous studies illustrating that between the ages of 4 and 5, children begin to take into account the recipient’s identity (e.g., whether the recipient was a friend, familiar non-friend or stranger of the distributor) when anticipating sharing and prosocial behavior in both first-person (Moore, 2009) and third-party (Paulus and Moore, 2014) contexts. Thus, in addition to social relations involving friendships (Paulus and Moore, 2014) and dominance structures (Enright et al., 2017), parent-child relationships also appear to be a type of social relationship that influence children’s fairness expectations. The findings also provide insight into children’s fairness expectations by highlighting that in addition to merit (Baumard et al., 2012) and need (Paulus, 2014)—two well-known factors influencing children’s expectations for equality—relationship-type also appears to be a factor that alters children’s egalitarian preferences by leading to expectations for partiality in parent-child relationships.

### Developing an understanding of kin relationships: Are parent-child relationships special?

It is important to note that children’s ability to use partiality as a cue to infer and understand third-party social affiliations may not be exclusive to parent-child relationships. Indeed, partiality may be a cue used to determine the closeness of relationships (as opposed to serving as a cue to any specific type of relationship *per se*). Despite previous studies illustrating children’s abilities to use the observation of partiality to infer third-party friendships (Liberman and Shaw, 2017), our findings show that partiality cues are not *only* used to infer third-party friendships, but also extend to other types of relationships as well, such as parent-child relationships. Critically, the findings from the current study move the field forward in two key ways: (1) by illustrating that children link partiality and third-party social affiliations in a *bidirectional* manner, and (2) that the presence/absence of these cues (partiality and parent-child relationships) also affect children’s social *evaluations*.

Another important question for future work is whether the ability to infer parent-child relationships develops in advance of, or alongside, other types of kinship relationships. Previous work has found that children demonstrate an explicit understanding of sibling relationships by the age of 5, such that children hold different expectations regarding sharing and prosociality for kin compared to non-kin relationships (Spokes and Spelke, 2016). In a study conducted by Spokes and Spelke (2016), participants were presented with hypothetical scenarios and asked whether a protagonist character would help (e.g., by completing a puzzle) a sibling or a different child (i.e., a stranger or friend). Five-year-old participants anticipated preferential help to be displayed towards siblings versus strangers and siblings versus friends, however, 3- and 4-year-old participants did not differentiate between different types of close interpersonal relationships (i.e., between a sibling or a friend). This study suggests that it is not until the age of 5 in which children are able to understand cues related to sibling relationships.

The fact that children in the present study understood third-party parent-child relationships by the age of 4, can be interpreted in various ways. First, these findings raise the possibility that children’s ability to use cues to specify parent-child relationships may precede their ability to identify sibling relationships or other types of kin relationships. Characteristically, parent-child relationships are distinct from other types of kin relationships in a number of key ways (e.g., Russell et al., 2002). Parent-child relationships are asymmetrical in nature, such that parents are expected to care and provide resources for their children (Kaplan et al., 2000; Keller, 2000), whereas children are not expected to reciprocate these actions towards their parents (at least not in early childhood; Russell et al., 2002). This is in stark contrast to other types of kin relationships, such as sibling relationships, that are characterized as horizontal in nature and operate based on notions of reciprocity (Russell et al., 2002). Further, parents are fundamental in children’s survival, as parents provide their children with access to food and shelter (Trivers, 1972; Kaplan et al., 2000; Keller, 2000). Thus, given the unique structure of parent-child relationships, as well as the fundamental role that parents have in the survival and well-being of their offspring (Kaplan et al., 2000; Keller, 2000), it is possible that a developmental decalage exists in children’s abilities to identify particular types of kin relationships, and that the ability to identify parent-child relationships may be developmentally primary.

An alternate, but not mutually exclusive, possibility is that the type of social cue examined, partiality in resource distributions, is more strongly associated with and/or more frequently experienced in parent-child compared to sibling relationships. Whereas partiality in resource sharing from parent to child is likely common, given parents’ drive to invest in their children to help ensure their survival (Trivers, 1972; Kaplan et al., 2000; Keller, 2000), sibling relationships are often more strongly characterized by reciprocity and a ‘tit-for-tat’ exchange (Russell et al., 2002). Therefore, displays of partiality in resource distribution towards siblings may be less frequent and/or more contingent on prior behavior. As such, it is possible that particular types of social cues, such as partiality in resource distributions, are more strongly associated with certain types of kin relationships, such as parent-child relationships, compared to others.

Finally, children’s “precocious” performance on the parent-child tasks versus sibling tasks may have to do with the fact that our parent-child tasks were arguably simpler compared to previously employed sibling tasks. For example, one feature that may have made the current study simpler compared to that of Spokes and Spelke (2016) was that participants were presented with a forced-choice task in which the participants were asked to categorize the adult as the mother versus not the mother (Kinship task), as opposed to disentangling behavioral expectations for various types of close interpersonal relationships (i.e., comparing siblings versus friends versus strangers; Spokes and Spelke, 2016).

Future work can directly compare the developmental trajectory of children’s abilities to understand the relationship between partiality and parent-child relationships versus partiality and sibling relationships, by using carefully matched tasks and comparisons. By comparing these two types of relationships, insight will be provided as to whether there is a developmental decalage in children’s ability to identify and understand different kin relationships or whether there is no such decalage but differences in task-related factors that account for such differences across studies. Currently, it remains an open question as to whether or not children’s understanding of various types of familial relationships follows different or similar developmental trajectories.

### Partial resource sharing as a cue to parental investment

Partiality in resource sharing from adult to child may be a particularly potent cue to parent-child relationships in third-party contexts because partiality may serve as an index of parental investment. Theories have postulated that one of the primary cues that individuals use to aid in first-person kinship detection is the observation of parental investment, more specifically, the observation of maternal perinatal association (e.g., observing a woman breastfeed an infant; Lieberman et al., 2007). Consistent with these claims, parents report more willingness to invest resources (e.g., financial assistance; Anderson et al., 1999) towards their own child as opposed to other children. Parents’ willingness to invest in their own children through the allocation of food, shelter, energy and time can be viewed as an evolutionary adaptive mechanism designed to aid in the survival and reproduction of their offspring (see Parental Investment Theory; Trivers, 1972). To the extent to which children use partiality in resource distributions as a means to signal parental investment, other forms of selective or pronounced resource sharing should be equally potent for specifying parent-child relationships in third-party contexts.

Another way in which parents invest in their children is *via* altruistic acts; for example, parents will often report more willingness to undertake costly acts to help support their child, by serving jail time on behalf of the child, by donating a kidney to help save the child, and by providing financial assistance for the child, when compared to unrelated, or even less closely related, children (Antfolk et al., 2017). Indeed, a recent study by Mammen et al. (2021) provides evidence that children more readily anticipate costly altruistic acts from parents versus peers. Mammen et al. (2021) asked participants to determine whether a parent or peer would act altruistically towards a child, and found that children as young as 6 expect parents (but not peers) to engage in costly acts, such as giving up their water for a thirsty child or giving their scarf to a child who is going out into the cold. These findings dovetail with the current study, in that they demonstrate that it is by this same age that children have greater expectations of partiality in resource distributions toward the target child when an adult is the child’s mother versus neighbor. Of course, the current study also demonstrated that children as young as 4 can make the reverse inference (i.e., use partiality to infer a parent-child relationship); thus, of interest is whether children under the age of 6 can use the presence/absence of costly giving to infer whether an adult is a child’s mother or not. Addressing this question will be an important means of testing whether children more broadly use features of resource sharing to signal parental investment, and thus rely on these cues to identify parent-child relationships.

## Conclusion

The current study provides novel evidence that partiality is a valuable cue to aid in third-party parent-child kinship detection, starting from age 4. The findings from the current study help to inform theories of fairness and equality by illustrating that although children hold expectations for equality, these expectations alter based on knowledge of close interpersonal relationships, in particular parent-child relationships.

Viewing partiality as a form of parental investment provides a theoretical framework through which the findings from the current study can help inform our understanding of third-party kinship detection. The findings from the current study illustrate that children expect parents to invest in their children, and that they use the observation of parental investment as a cue to aid in identifying third-party parent-child relationships.

## Data availability statement

The raw data supporting the conclusions of this article will be made available by the authors, without undue reservation.

## Ethics statement

The studies involving human participants were reviewed and approved by University of Toronto Ethics Board. Written informed consent to participate in this study was provided by the participants' legal guardian/next of kin.

## Author contributions

AM and JS contributed to the conception and design of the study. AM and SB organized the database. AM performed statistical analyses and wrote the first draft of the manuscript. SB wrote a section of the manuscript. AM, SB, MS, and JS contributed to edits of the manuscript. All authors contributed to the article and approved the submitted version.

## Funding

The current research was funded by NSERC Discovery Grant awarded to MS and a grant from the John Templeton Foundation awarded to JS.

## Conflict of interest

The authors declare that the research was conducted in the absence of any commercial or financial relationships that could be construed as a potential conflict of interest.

## Publisher’s note

All claims expressed in this article are solely those of the authors and do not necessarily represent those of their affiliated organizations, or those of the publisher, the editors and the reviewers. Any product that may be evaluated in this article, or claim that may be made by its manufacturer, is not guaranteed or endorsed by the publisher.

## Footnotes

^1^ https://www.pixton.com

^2^ https://apps.apple.com/ca/app/notability/id360593530

^3^Analyses for the forced-choice task were conducted with the inclusion and removal of participants’ responses who incorrectly answered the quantity questions (7.4% of trials). The patterns of results did not significantly differ with the inclusion or exclusion of these participants’ responses on the forced-choice task and as such, all forced-choice responses (even participants who answered incorrectly to the quantity questions) were included in the final analyses.

^4^There were small deviations from protocol in the administration of the Kinship task. When responding to the forced-choice question, "Is this the mother or not the mother?" some participants simply responded by saying, "Yes," or "No." Experimenters were asked to clarify these responses by repeating the forced-choice question and to not infer that the child meant, "Yes-the mother," or "No-not the mother." However, on a small subset of trials (7.4%) the experimenter failed to ask follow-up clarification questions. The analyses were conducted with these assumptions as part of the dataset and compared to analyses that excluded these assumptions. The overall patterns of findings did not differ between the two analyses. As such, all responses were included in the final analyses.

^5^There was a small difference in how the two experimenters administered the Emotion task. If the participant selected an equitable distribution of resources in the Resource Distribution task, one experimenter followed up by asking the participant how the protagonist would feel as a result of the equitable distribution. In contrast, the other experimenter followed up this scenario by asking the participant how the protagonist would feel if the adult (either mother or neighbor) distributed the resources equitably. In essence, the second experimenter reiterated the adult character’s identity, whereas the first experimenter did not. Analyses were run with experimenter as a between-subjects variable. No significant effect of experimenter emerged. As such, the results are presented without the inclusion of experimenter as a variable.

^6^ https://aspredicted.org/blind.php?x=6um5g6

^7^For the analyses for the Resource Distribution task, one participant from the 4- to 5-year-old age group was removed due to an ambiguous answer from the participant that was not clarified by the experimenter.

^8^For the analyses of the Emotion task, the sample size consisted of 65 participants, instead of 68. Thirty-two 4- to 5-year-olds and 33 6- to 7-year-olds were included in the final sample.
